# Patient-Tailored Therapy for Complex Aortic Arch Anatomy: An Evolving Research Field with Custom-Made Solutions

**DOI:** 10.3390/jcm13174975

**Published:** 2024-08-23

**Authors:** Daniele Linardi, Jacopo Gardellini, Vincenzo Boschetti, Venanzio Di Nicola, Mariateresa Denora, Gino Puntel, Giovanni Puppini, Giovanni B. Luciani

**Affiliations:** 1 Cardiac Surgery Department, Azienda Ospedaliera Universitaria Integrata Verona, 37126 Verona, Italygiovanni.luciani@univr.it (G.B.L.); 2 Radiology Department, Azienda Ospedaliera Universitaria Integrata Verona, 37126 Verona, Italy

**Keywords:** tailored therapy, aortic team, aortic arch treatment

## Abstract

The treatment of complex aortic pathologies requires specialized techniques and tailored approaches due to each patient’s unique anatomical and clinical challenges. The European Association for Cardiothoracic Surgery (EACTS) and the Society of Thoracic Surgeons (STS) new guidelines identify the aorta as the body’s 24th organ and reiterate that multidisciplinary aortic teams are recommended for shared decision-making to determine optimal treatment strategies. Patients treated for conditions such as aneurysms, dissections, intramural hematomas, or penetrating aortic ulcers may develop complex forms over time, necessitating careful follow-up and timely corrective actions. Endovascular solutions can be favorable for older patients with complex anatomies and multiple comorbidities. However, when endovascular treatment is not feasible, hybrid treatments or open surgery must be considered if the patient’s condition allows it. The risk–benefit ratio of each procedure must be carefully evaluated; choosing the best intervention or deciding not to intervene becomes a critical and challenging decision. At our Cardiac Surgery Center in Verona, a multidisciplinary team with over 20 years of experience in treating complex aortic arch pathologies extensively discussed different cases of complex aortic pathologies treated with endovascular, hybrid, or surgical approaches, emphasizing the importance of considering both anatomical and patient-specific characteristics. The decisions and treatments were often challenging, and unanimity was not always achieved, reflecting the complexity of finding the best solutions.

## 1. Introduction

The treatment of complex pathologies is not straightforward and may require a more patient-specific approach based on different available techniques.

The presentation may be in an acute setting or a referral following previous surgical or endovascular intervention requiring multidisciplinary input, preferably in designated high-volume aortic centers. Shared decision-making for the optimal treatment of aortic pathologies by a multidisciplinary aortic team is recommended [1].

Older patients who were treated at a younger age for conditions such as aneurysms, type A or B dissections, intramural hematomas (IMH), or penetrating aortic ulcers (PAU) may experience dilated evolutionary changes with unpredictable dynamics. Therefore, careful consideration must be given to follow-up and the timing of corrective actions. As the patient population increasingly consists of elderly individuals with multiple comorbidities, determining the most appropriate intervention requires careful reasoning. In some cases, the decision not to intervene might be the best course of action, though it is always challenging for a physician to accept being incapable of offering a solution for a patient with a predictable destiny [2].

When dealing with complex anatomies in patients with multiple comorbidities, an endovascular approach may often be the most appropriate solution due to its minimal impact on the patient [3]. However, there are instances where endovascular treatment is either not feasible or not the best option. In such cases, hybrid treatments or open surgery must be considered, provided the patient is in a suitable condition. It is the responsibility of the treating physician to carefully assess the risk–benefit ratio of each procedure.

This manuscript presents some examples of complex aortic pathologies managed through endovascular, hybrid, or open procedures, with careful consideration given to both the anatomical features of the aorta and the patient’s individual characteristics.

In our Cardiac Surgery Center in Verona, with over 20 years of experience in treating complex pathologies of the aortic arch, each case was thoroughly discussed by our multidisciplinary thoracic aortic pathology team. The final decisions and treatments were not always reached by unanimous agreement, reflecting the challenges in determining the best approach.

## 2. Case Presentation

### 2.1. Patient 1

A 73-year-old lady presented to A&E with a sudden onset of chest pain radiating to the back in the context of hypertension, multi-nodular goiter, psoriatic arthritis, nephrolithiasis, and a history of deep vein thrombosis and pulmonary embolism. Computed Tomography Angiography (CTA) revealed a PAU with saccular pseudoaneurysm formation (24 × 35 mm) on the left aspect of the aortic arch. The position of the aortic lesion was not favorable, as it corresponded with the origin of the left carotid artery. Clinical presentation confirmed the sub-acute onset and progression of the pathology. Additionally, the patient was under follow-up for multi-nodular goiter, with a planned surgical treatment involving total thyroidectomy. Different strategies were considered, with open chest surgery deemed too risky for the patient. An alternative surgical approach involving the creation of supra-aortic branch bypasses and the placement of an endovascular aortic prosthesis could complicate the multi-nodular goiter surgery. Therefore, total endovascular treatment was considered the optimal strategy [4].

**Figure 1:**
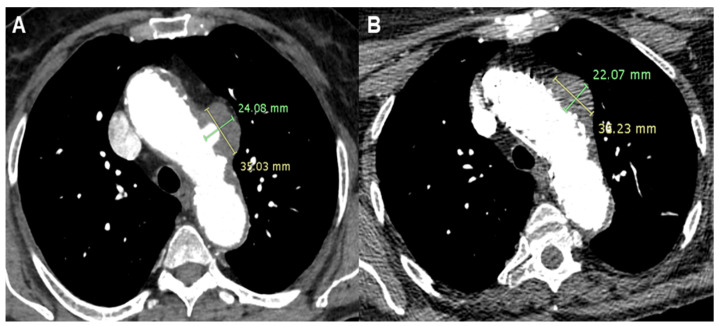
Patient 1’s Computed Tomography Angiography showing a Penetrating Aortic Ulcer (PAU) of the aortic arch (**A**) and the complete exclusion of the PAU after Thoracic Endovascular Aortic Repair (TEVAR) with reduction in the dimension of the pseudoaneurysm (**B**).

Following a period of clinical observation without further episodes of chest pain, a customized cone-shaped prosthesis was designed to accommodate the proximal aortic arch dilatation. It featured a scalloped design to fit the origin of the left carotid artery and included a fenestration for the left subclavian artery (Terumo, Custom Relay Plus 38-32 × 180 mm). A stent was placed at the origin of the left subclavian artery and inserted through the fenestration to ensure branch patency. Additionally, to ensure proper prosthesis adaptation and prevent potential type I endoleak formation, endovascular ballooning with transvenous rapid cardiac pacing was performed to seal the proximal end of the aortic prosthesis in Ishimaru Zone 1 (Figure 1).

### 2.2. Patient 2

A 77-year-old gentleman with an aortic arch aneurysm underwent Thoracic Endovascular Aortic Repair (TEVAR) in Ishimaru Zone 1 and concomitant extra-anatomical bypass between the right and left common carotid and left subclavian arteries in 2011. He was subsequently lost at follow-up, although he turned up years later with an imaging investigation showing a type I endoleak in the context of a severe pseudoaneurysm formation, 72 × 130 mm, expanding around the aortic arch. A rich medical history developed over the years made the condition more challenging to treat. Endovascular intervention was considered more appropriate compared to a high-risk surgical procedure with significant potential for a fatal outcome. Given that cerebral circulation was sustained by the single innominate artery, managing this branch was crucial in planning the intervention. A prosthesis was required to ensure adequate flow through the supra-aortic branch and address the endoleak while fitting the anatomy of the aortic arch [5]. TEVAR with a custom-made prosthesis, featuring a proximal landing zone in Zone 0, an inner branch for the innominate artery, and concomitant stenting of the artery itself, was the chosen approach (Terumo, Custom Relay Pro 42-36 × 180 mm).

**Figure 2:**
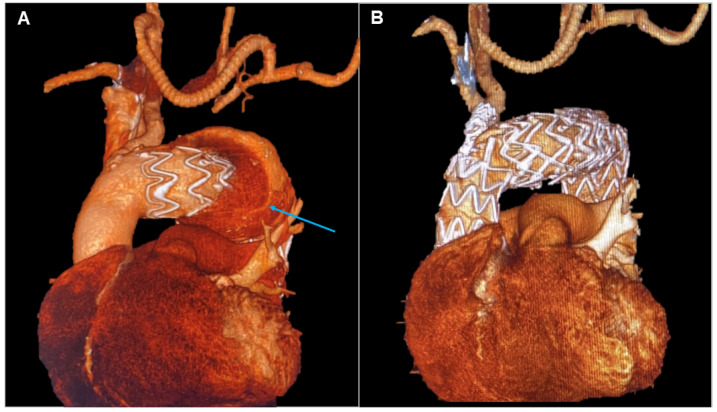
Patient 2’s 3D reconstruction showing the right common carotid–left common carotid and left common carotid–left subclavian artery bypass and the type IA endoleak (blue arrow) (**A**). In panel (**B**), the result of the treatment with a custom-made solution with a landing zone in Zone 0 and a single branch for the innominate artery.

The wide proximal landing zone in a non-dilated ascending aorta effectively blocked blood leakage from the pseudoaneurysm. Meanwhile, stenting of the innominate artery ensured patency of the supra-aortic branches and prevented potential new endoleaks through the prosthesis fenestration. Post-procedure CTA demonstrated complete endoleak exclusion, with normal vascularization of the supra-aortic branches and no evidence of additional endoleaks, resulting in a positive outcome for the patient (Figure 2).

### 2.3. Patient 3

**Figure 3:**
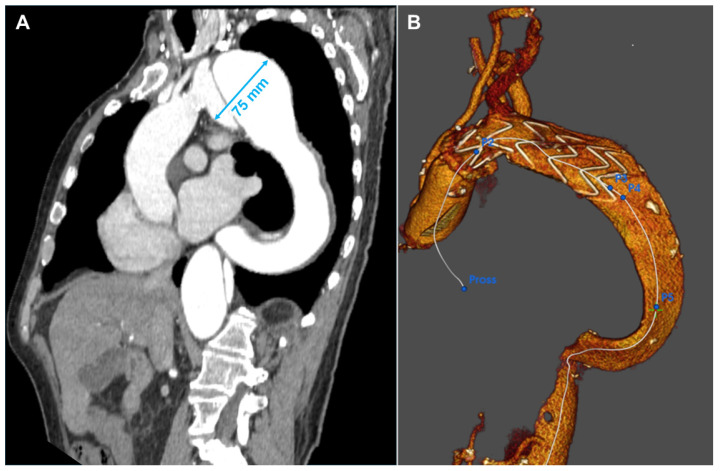
Patient 3’s Computed Tomography Angiography showing the evolution of the type B dissection with an enormous aneurysm of 75 mm in Zone 3 (**A**). Three-dimensional reconstruction of the result after the frozen elephant trunk operation (**B**). After one month, the patient was treated with another TEVAR in the descending aorta.

A 66-year-old gentleman presented with an aneurysm of 75 mm of the distal aortic arch and proximal descending thoracic aorta just below the origin of the subclavian artery, in the context of chronic type B aortic dissection previously managed conservatively. Further dilatation of the descending thoracic aorta with kinking at the diaphragm made the anatomy more challenging. Aneurysmatic dilatation of the ascending aorta precluded an endovascular option alone. Therefore, aortic arch replacement was considered a suitable approach [6]. A left common carotid–left subclavian artery extra-anatomical bypass was performed, followed by a frozen elephant trunk (FET) with Jotec E-Vita Open Neo 30-120 × 30 prosthesis 24 h later. The innominate and left common carotid arteries were re-implanted. Finally, significant disease of the left anterior descending coronary artery required grafting with the left internal mammary artery. The patient’s aortic pathology was further complicated by severe peripheral vascular disease, including total occlusion of the left external iliac artery and the right common iliac artery originating from the false lumen. Despite the unfavorable anatomy, a TEVAR (Terumo Relay Pro 32 × 32 × 155 mm) was performed, extending the distal landing zone to prevent caudal endoleak formation and exclude the aortic aneurysm. To address the type II endoleak originating from the left subclavian artery, a vascular plug was placed at its aortic origin, which completed the false lumen thrombosis of the Zone 4 aneurysm and the descending aorta. After a long and complicated postoperative course, the patient was discharged and continued with regular CTA follow-up, demonstrating clinical and anatomical stability (Figure 3).

### 2.4. Patient 4

An 85-year-old gentleman underwent left common carotid–left subclavian artery extra-anatomical bypass followed by TEVAR for a distal aortic arch aneurysm in 2014. Subsequently, the development of a type IB endoleak required further TEVAR in 2019. Then, coil embolization was required for a type IA endoleak in 2020. Nevertheless, follow-up imaging investigation showed further development of type IA endoleak up to 83 mm in diameter with significant dilatation of the proximal aortic arch and aneurysm of the ascending aorta at 58 mm in diameter. The echocardiographic assessment revealed severe aortic regurgitation. Significant comorbidities included previous myocardial infarction requiring percutaneous intervention and stenting of the right coronary artery, chronic renal impairment, previous neurological event, giant cell arteritis, and rheumatological disease. The presence of aortic valve disease and the severity of the aortic arch aneurysm made a total endovascular approach an unsuitable option. In contrast, the patient’s comorbidities made total aortic arch replacement quite a high-risk surgical procedure. 

**Figure 4:**
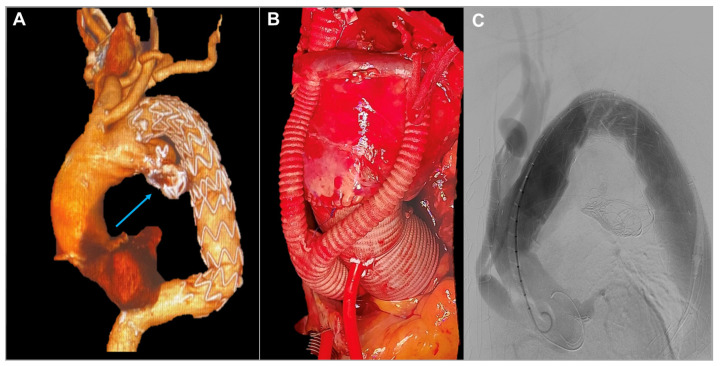
Patient 4’s 3D reconstruction showing the previously positioned TEVAR with left carotid–left subclavian artery bypass, the truncus bovinus, and the type IA endoleak (blue arrow) (**A**). Intraoperative image of the ascending aorta replacement and the debranching with the division of the left carotid artery from the innominate artery (**B**). Angiography of the final result after Zone 0 landing of the TEVAR with complete exclusion of the endoleak (**C**).

The patient had repeatedly refused surgery in agreement with the treating doctors. However, after multiple episodes of chest pain, the patient and his family insisted on proceeding with the operation, despite the high operative risk. A hybrid approach was planned, involving aortic valve and ascending aorta replacement with debranching of the supra-aortic branches (Edwards Perimount Magna 25 mm, Vascutek Gelweave 34 mm, Hemagard Knitted 16-8 mm) to ensure a proper proximal landing zone for subsequent endovascular treatment of the arch lesion [7]. A few days later, a TEVAR (Terumo Relay Pro 42-42-200 mm) was performed, and the endovascular prosthesis was deployed in Ishimaru Zone 0. This approach minimized intraoperative risks, ensured cerebral perfusion through supra-aortic debranching, and achieved type IA endoleak sealing (Figure 4).

Despite the absence of neurological events, the postoperative course was complicated by prolonged mechanical ventilation followed by percutaneous tracheostomy. Subsequently, a slow but steady weaning from the ventilator with aggressive intensive care treatment led to transfer to the peripheral hospital for continuity of care. Sadly, a bacterial infection resulted in a fatal outcome 30 days after surgery [8,9].

### 2.5. Patient 5

A 78-year-old gentleman underwent TEVAR in 2002 for a post-traumatic saccular pseudoaneurysm. In April 2021, he was diagnosed with severe aortic valve stenosis. He underwent an electrocardiogram (ECG)-gated CT study to plan a trans-apical aortic valve implant, which also revealed a perfused, ulcerated plaque outside the endoprosthesis consistent with a type IB endoleak. We prioritized aortic valve implantation and planned follow-up CTA of the thoracic aorta after the aortic valve treatment [10]. Postoperatively, during cardiological rehabilitation, the patient contracted SARS-CoV-2, which required hospitalization and a prolonged stay in the ICU. Two months after surgery, another ECG-gated CT was performed due to a suspected left auricle thrombus during hospitalization. The scan revealed a completely new scenario with post-isthmic aneurysmal dilatation and a large type III endoleak (65 mm) with a thick eccentric thrombotic flap. There was also posterior expansion causing erosion of the lateral walls of C5 and C7.

A few days later, a TEVAR was performed (Terumo Relay Pro 34-34-200 mm) with the proximal landing just after the origin of the left subclavian artery. Follow-up CTA showed a reduction in the axial diameter of the aneurysm (61 mm vs. 65 mm) and a marked reduction in the previously observed endoleak phenomena. The postoperative course was uneventful, with the patient’s ICU stay lasting one day and discharge occurring six days after the procedure.

**Figure 5:**
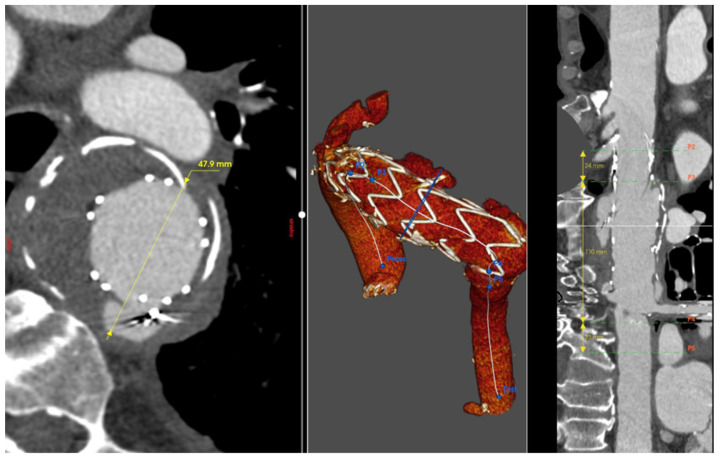
Patient 5’s Computed Tomography Angiography reconstruction showing the type III endoleak caused by rupture of the TEVAR secondary to infection.

Three months following TEVAR, the patient developed oral bleeding. Fibro-laryngoscopy revealed diffuse mucous varicose veins in the nose and at the base of the tongue. Surgery was performed to cauterize the varicose veins at the tongue base. Follow-up CTA showed an increase in the diameter of the excluded aneurysm with the appearance of type II endoleaks, as well as some periprosthetic air traces and suspected aorto-bronchial fistula (Figure 5). Given the clinical situation, the patient was readmitted for further diagnostic evaluation. No macroscopic mucosal fistulous pathways were observed via gastroscopy or tracheobronchoscopy. Blood cultures and PET-CT were conducted, revealing periprosthetic uptake suggestive of an infectious process. Concurrently, likely due to antibiotic therapy and various diagnostic procedures, the patient developed acute renal failure, ultimately requiring dialysis.

After extensive discussion of the clinical case, descending aorta replacement was decided against due to the high operative mortality risk. The patient was discharged to a peripheral hospital, where he continued dialysis and antibiotic therapy. Unfortunately, the patient’s condition progressively worsened, and he passed away approximately eight months after the last TEVAR procedure [11].

## 3. Discussion

**Figure 6:**
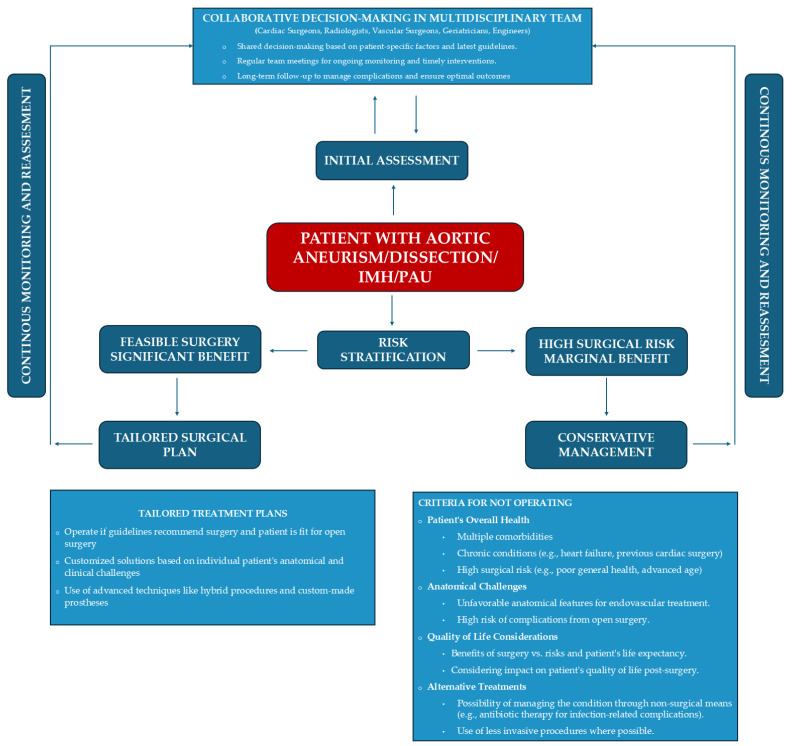
Decision-making flowchart for the correct treatment of the patient with complex aortic pathology. The patient is at the core of the decision-making process, where the multidisciplinary team carefully assesses all patient-specific factors to determine the most effective treatment strategy.

Surgical treatment of aortic arch aneurysms remains controversial due to concerns about the effectiveness of cerebral protection, the need for hypothermic circulatory arrest, and the risk of bleeding. The operative risk is further amplified in cases involving associated cardiac pathologies, previous aortic surgery, or multiple comorbidities specific to the patient. As a result, endovascular approaches or less invasive hybrid procedures (combining surgery with endovascular treatment) have been explored to improve survival and reduce operative risk. Nevertheless, selecting the most appropriate procedure for each patient is not always straightforward (Figure 6).

In Patient 1, the treatment options could include three approaches: surgical, hybrid with a type I debranching or carotid–carotid–subclavian bypass combined with endoprosthesis coverage up to Ishimaru Zone 1, or the use of a custom endoprosthesis [12]. An immediate approach involving bypass and endoprosthesis could have been implemented, potentially avoiding the risks of new onset chest pain or rupture of the pseudoaneurysm originating from the PAU. However, the presence of a large thyroid goiter, scheduled for surgical removal, led us to pursue a fully endovascular approach. The risks associated with this decision were communicated to the patient and her family. The surgery was delayed, allowing engineers to design and fabricate a custom-made endoprosthesis. The outcome was excellent, even with the presence of a type I endoleak, which was promptly addressed. This case underscored the importance of patient-tailored solutions, highlighting that the optimal outcomes often require more time to design and implement compared to traditional, immediately available solutions [13].

In Patient 2, both open surgery and endovascular procedures posed a high neurological risk due to the presence of a single vessel supplying blood to the brain [14]. Discussions with radiologists and engineers led to the development of a custom-made prosthesis designed to minimize impact on the patient, who was 11 years older than during the first procedure. Implanting an endoprosthesis in Ishimaru Zone 0 is not without risks. For improved proximal sealing, the endoprosthesis is deployed during rapid pacing. Additionally, positioning the inner branch for the innominate artery requires the placement of stents and balloon inflation, which temporarily obstructs the sole inflow for cerebral perfusion [15]. In contrast, an open procedure would involve aortic arch surgery with cardiopulmonary bypass using arterial access from the right axillary artery to maintain cerebral perfusion during circulatory arrest. It is challenging to predict which of the two approaches would be riskier neurologically. However, for this 78-year-old patient, the endovascular approach offered a quicker recovery and reduced hospitalization, with the patient being discharged on the fifth postoperative day. In this context, the cost of the custom-made endoprosthesis was justified by the benefits of a shorter ICU stay and reduced overall hospitalization, which minimized the risk of additional complications such as prolonged ventilation, the need for cardiovascular rehabilitation, or sternal wound dehiscence [16].

In Patient 3, the considerable size of the descending aortic aneurysm necessitated a prompt, multi-stage hybrid approach. Initially, a carotid–subclavian bypass was performed, followed by a frozen elephant trunk procedure with anastomosis in Zone 2 and subsequent TEVAR of the descending aorta [17]. The advanced vascular disease significantly complicated the treatment, reducing accessibility and increasing the patient’s risk. Peripheral vasculopathy further complicated the procedure, making femoral artery access for TEVAR challenging. The total occlusion of the left iliac artery required advancing the endoprosthesis from the false lumen to the true lumen to achieve proper positioning. The patient endured a complex postoperative course, including four surgeries, approximately two months of hospitalization, and an additional month of rehabilitation.

In this scenario, endovascular treatment alone was not feasible, necessitating a surgical approach. Anatomical factors, such as the size of the aortic aneurysm and the unfavorable angle of the aortic arch, can complicate the deployment of an endovascular prosthesis. Aortic surgery offers a range of strategies, from hybrid approaches to open chest surgeries. When a longer proximal landing zone is required, aortic debranching can be an effective strategy, enabling successful endovascular treatment even up to Ishimaru Zone 0.

Patient 4 was an elderly man with multiple comorbidities, chronic corticosteroid use, and ascending aortic dilatation that precluded endovascular treatment, necessitating at least an ascending aorta replacement. This patient’s case was extensively discussed within the multidisciplinary team. While a FET could have been a comprehensive strategy, the intervention was ultimately deemed too burdensome for him. The patient underwent a hybrid treatment involving central debranching and endovascular procedures the following day. The final angiography revealed a small type I endoleak, which was decided to be monitored over time [18]. Unfortunately, the patient never left intensive care; extubation proved impossible, and his condition deteriorated further due to an intra-hospital infection, leading to his death about a month later. In this case, the hybrid approach was the least invasive option and seemed most appropriate given the patient’s request to perform surgery due to recurrent episodes of chest pain. The question arises whether it would have been justifiable to refuse the intervention or to deem the patient inoperable. Deciding not to operate can be one of the most challenging decisions. However, a multidisciplinary team must recognize when forgoing intervention might be the best choice to ensure the patient’s remaining time is of the highest possible quality. In this regard, involving a specialist geriatrician or a dedicated internal medicine specialist in the aortic team could help to better define the patient’s risk profile and guide such difficult decisions.

Patient 5 developed a severe complication 20 years after the initial endovascular repair of a post-traumatic saccular pseudoaneurysm. The treatment performed in 2002 was appropriate for the time and should not be questioned; it was known by our engineering team that the type of endoprosthesis used could potentially degenerate with tissue rupture after many years. However, it later became clear that an infection had precipitated the rupture. The incidence of infection around the graft, extending to the esophagus, has been reported to be between 0.5% and 5% [19]. The correct course of action in such a scenario would have been to replace the descending aorta. However, this procedure carries a high mortality and morbidity risk, so the team opted to try extending the patient’s life through lifelong antibiotic therapy [20]. Unfortunately, the patient never recovered from acute renal failure and remained hospitalized, continuing antibiotic treatment at a peripheral hospital.

The occasional episodes of hemoptysis were indicative of a high risk of aortic rupture, even though an aortic–bronchial fistula was never conclusively diagnosed through PET-CT or bronchoscopy. In this case, was the decision not to intervene the right one? Could a chance of survival have been offered through high-risk surgery?

Despite the potential benefits of patient-tailored treatments, several challenges remain, including the limited availability of high-quality evidence, variability in expertise, and differences in resource availability. Future research should prioritize prospective studies to validate risk stratification tools, refine treatment algorithms, and assess long-term outcomes. Minimally invasive techniques, such as TEVAR, offer significant advantages for patients with multiple comorbidities. As technologies evolve, so do indications and guidelines, which are becoming increasingly refined. While treatment decisions are more straightforward for older patients, the best approach for younger patients remains a subject of ongoing debate.

For younger patients with longer life expectancy, the choice of surgical or interventional technique must be made with great care, taking into account the individual patient’s condition and weighing the potential benefits and risks associated with either an aggressive surgical approach or a more conservative strategy with ongoing follow-up [21]. The debate continues around the surgical or endoprosthetic treatment of the aortic arch, particularly in determining how aortic pathologies will evolve years after the placement of an endoprosthesis. Continuous follow-up is crucial throughout the patient’s life, with the frequency determined by radiological and clinical findings. This vigilance is essential to prevent significant complications or the unchecked progression of aortic pathology.

Complex aortic pathologies demand a multidisciplinary approach, drawing on diverse expertise to ensure optimal patient care. Each team member contributes unique skills and knowledge, fostering a comprehensive understanding of the patient’s condition and the most suitable treatment options. The multidisciplinary team undertakes thorough risk stratification, considering patient-specific factors and the latest evidence-based guidelines. This approach aids in identifying patients at higher risk for adverse events, allowing the team to develop strategies to mitigate those risks and safeguard patient safety throughout the treatment process [22]. Aortic pathologies often require long-term management and follow-up. A multidisciplinary team ensures continuity of care by coordinating preoperative evaluation, surgical interventions, postoperative care, and long-term surveillance. Regular team meetings and discussions facilitate ongoing monitoring of the patient’s progress, enabling the early detection of complications and the timely implementation of necessary interventions.

## 4. Conclusions

The cases discussed illustrate both the successes and challenges in treating complex thoracic aortic pathologies. While the literature often highlights clinical successes through case reports, the failures of proposed treatments deserve more attention in scientific meetings and journals. This openness is crucial for the growth of future professionals, who must learn from both successes and failures. The establishment of multidisciplinary aortic teams—comprising cardiac surgeons, radiologists, vascular surgeons, geriatricians, and internists—plays a pivotal role in considering all of the patient’s conditions and addressing therapeutic challenges.

Choosing the most effective treatment for a specific pathology in a particular patient embodies the principles of patient-tailored medicine. Each clinical case is unique, and a multidisciplinary expert team is essential for optimal outcomes. Such teams bring together diverse expertise, facilitate collaborative decision-making, tailor treatment plans, ensure patient safety, and promote continuity of care. Their collective efforts significantly enhance the quality of care provided to patients with complex thoracic aortic conditions, leading to better outcomes and greater patient satisfaction.

## Data Availability

Data and imaging regarding the cases presented can be requested from the corresponding author.
